# Impact of obesity and other risk factors on labor dystocia in term primiparous women: a case control study

**DOI:** 10.1186/s12884-018-1938-3

**Published:** 2018-07-18

**Authors:** Tuija Hautakangas, Outi Palomäki, Karoliina Eidstø, Heini Huhtala, Jukka Uotila

**Affiliations:** 1 Department of Obstetrics and Gynecology, Central Hospital of Central Finland, Keskussairaalantie 19, 40620 Jyväskylä, Finland; 2 Department of Obstetrics and Gynecology, Tampere University Hospital, Tampere, Finland; 3 Kangasala Health Center, Kangasala, Finland; 4 School of Health Sciences, Tampere University, Tampere, Finland

**Keywords:** Dystocia, Primipara, Obesity, Cesarean section, Case control

## Abstract

**Background:**

Purpose of this study was to investigate differences between primiparous term pregnancies, one leading to vaginal delivery (VD) and the other to acute cesarean section (CS) due to labor dystocia in the first stage of labor. We particularly wanted to assess the influence of body mass index (BMI) on CS risk.

**Methods:**

A retrospective case-control study in a tertiary delivery unit with 5200 deliveries annually. Cases were 296 term primiparous women whose intended vaginal labor ended in acute CS because of dystocia. Controls were primiparas with successful vaginal delivery VD (*n* = 302). The data were retrieved from medical records. Multiple logistic regression analyses were used to assess the associations between BMI and covariates on labor dystocia.

**Results:**

In the cases ending with acute CS, women were older (OR 1.06 [1.03–1.10]), shorter (OR 0.94 [0.91–0.96]) and more often had a chronic disease (OR 1.60 [1.1–2.29]). In this group fetal malposition (OR 42.0 [19.2–91.9]) and chorioamnionitis (OR 10.9 [5.01–23.6]) were more common, labor was less often in an active phase (OR 3.37 [2.38–4.76]) and the cervix was not as well ripened (1.5 vs. 2.5 cm, OR 0.57 [0.48–0.67] on arrival at the birth unit.

BMI was higher in the dystocia group (24.1 vs. 22.6 kg/m^2^, *p* < 0.001), and rising maternal pre-pregnancy BMI had a strong association with dystocia risk. If BMI increased by 1 kg/m^2^, the risk of CS was 10% elevated. Among obese primiparas, premature rupture of membranes, chorioamnionitis and induction of labor were more common. Their labors were less often in an active phase at hospital admission. Severely obese primiparas (BMI ≥ 35 kg/m^2^) had 4 hours longer labor than normal-weight parturients.

**Conclusions:**

Labor dystocia is a multifactorial phenomenon in which the possibility to ameliorate the condition via medical treatment is limited. Hospital admission at an advanced stage of labor is recommended. Pre-pregnancy weight control in the population at reproductive age is essential, as a high BMI is strongly associated with labor dystocia.

## Background

The rate of cesarean section (CS) has increased worldwide during the last few decades [1, 2] and even during the ongoing decade: the global CS rate in 2000–2008 was 13.9%, and it rose in 2007–2014 to 17%. In Europe as a whole the CS rate in 2007–2014 was 25% and in Finland it was 16% [1]. The CS rate has risen among both primiparous and multiparous women [2, 3]. The rate of intrapartum CS has increased significantly as well [2], the main indications being fetal distress and labor dystocia [4, 5].

Some risk factors of labor dystocia have been recognized or suggested. One of the contributors may be the increasing number of cases of labor induction [2, 3, 6]. A high body mass index (BMI) is associated with dystocia and intrapartal CS [7–9], as is advanced maternal age [3, 5].

Although CS can be a lifesaving operation, it can cause severe maternal morbidity, and sometimes lead to complications in the next pregnancy [10]. Thus, measures should be taken to reduce the number of unnecessary CSs, especially in primiparous women, as CS after the first pregnancy is prone to lead to repeat CSs in following pregnancies [2, 11].

The aim of this study was to assess the differences between two types of primiparous labor, one leading to vaginal delivery and the other to acute CS due to labor dystocia in the first stage of labor. We particularly wanted to investigate the influence of an overweight condition on the risk factors of labor dystocia.

## Methods

This was a retrospective parity-matched case-control study in a tertiary delivery unit (Tampere University Hospital) with an annual delivery rate of 5200. The study period was from February 2009 to December 2012. Data were obtained from medical files of the mother and newborn. Ethical approval for the study was given by the Ethics Committee of Pirkanmaa Hospital District (R12522S), 3rd April 2012.

The study group consisted of 296 term primiparous women whose intended vaginal labor ended in acute cesarean section as a result of dystocia, i.e. Robson groups 1 and 2A [12]. To obtain data for the study group, the birth register was searched for primiparas with WHO International Classification of Diseases (ICD)-10 diagnosis O82.10 (acute CS excluding emergency CS) with at least one of the dystocia-diagnoses O62, O63 or O64. One of the doctors form the study group checked the medical files of these primiparas to exclude the cases with twins and premature (< H37 + 0) labors. Pathological findings in cardiotocography (CTG) as the main indication for CS represented an exclusion criterion as was diagnosis of dystocia made after full dilatation of the cervix.

**Fig. 1:**
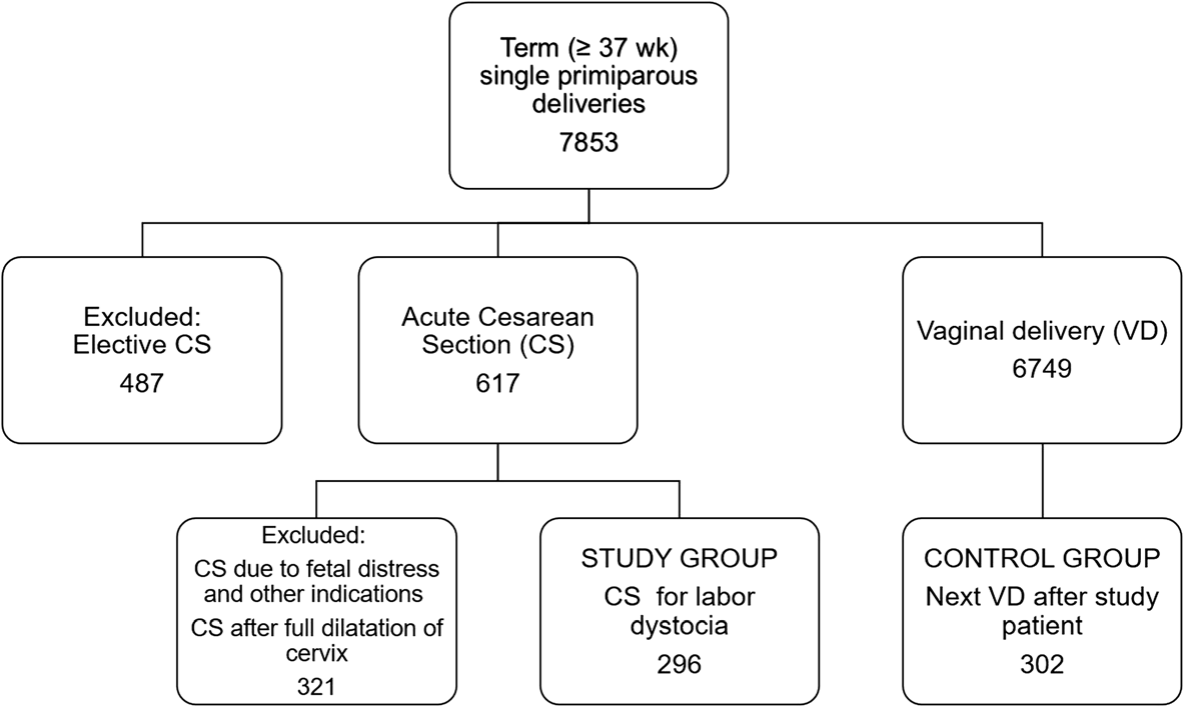
Number of singleton primiparous deliveries during the study period and the application of exclusion and inclusion criteria

Primiparous women at term, with vertex presentation and singleton pregnancies attending the delivery unit and with successful vaginal delivery were selected as controls (*n* = 302). Deliveries were spontaneous or augmented. A flow chart of the collected study cohorts is shown in Fig. 1.

Variables related to maternal background, labor characteristics and neonatal outcome were recorded and compared between groups. To study the effects of obesity on labor outcome and on the risk factors of dystocia, the parturients were divided into subgroups according to WHO BMI classification [13]. The weights of the primiparas were self-reported in the first antenatal visit in primary health care in gestational weeks 6 to 8. If there was discrepancy between self-reported and measured weight in this antenatal visit, or women were not aware of their pre-pregnancy weight, the weight measured by health professional was used. The BMI was calculated by the formula: weight (kg) ÷ height^2^ (cm). Almost every parturient was screened for gestational diabetes mellitus according to Finnish Current Guidelines during the pregnancy. The 2 h 75-g oral glucose tolerance test was made for all except the lowest risk primiparas (age under 25 years, BMI ≤ 25 kg/m^2^ or no family history of diabetes mellitus). If fasting blood glucose level was ≥5,3 mmol/l or 1 hour’s level ≥ 10 mml/l or 2 hours level ≥ 8,6 mmol/l, the diagnosis of gestational diabetes (GDM) was made. The fear of childbirth (FOC) was defined by a separate diagnose code used in Finland for that disorder in the medical records. All the parturients with this diagnosis had been visited maternity clinic of birth hospital to get treatment for FOC.

As our study was retrospective, it had not influence on the treatment of labor. Labors were treated by the hospital’s guidelines. During this period, a modified active management of labor- protocol was used and the maximum oxytocine dose limit was 15 mIU/min. Partograms were used in delivery rooms. Intrauterine pressure catheters were used, if external tocodynamometry did not provide enough information, and liberally among oxytocin augmented labors. Chorionamnionitis was defined by intrapartum temperature more than 38.0 or a combination of fetal tachycardia and maternal C-reactive protein more than 20 g/l.

All statistical analyses were performed using SPSS for Windows 23 (IBM SPSS Statistics for Windows, Version 23.0. Armonk, NY: IBM Corp.). Continuous variables were expressed as means with standard deviations, or medians with quartiles. Categorical variables were expressed as frequencies and percentages. Comparisons were made between the intrapartum CS and successful VD groups. The Mann–Whitney *U*-test, Fisher’s exact test and the chi-squared test were used, as appropriate. Associations between risk factors and the mode of delivery were analyzed separately. Multivariable analyses were performed separately as regards maternal background variables and intrapartum variables that were significant in univariate analyses. The results of logistic regression analyses were expressed as adjusted odds ratios (aORs) and 95% confidence intervals (95% CIs). A *p*-value of < 0.05 was considered statistically significant. All *p*-values were two-sided.

## Results

Table 1 shows maternal characteristics in the study groups. In multivariable analysis of the background variables, maternal age, height, BMI and chronic disease (e.g. asthma, thyroid, neurological or psychiatric disorders) remained as independent risk factors. Women in the dystocia group had a higher BMI, and if BMI increased by 1 kg/m^2^, the risk of CS was elevated by 10%. A 1 year increase in maternal age or a 1 cm decrease in maternal height increased CS risk by 6%.

**Table 1:** Maternal characteristics in the study groups

	CS (*n* = 296)	VD (*n* = 302)	univariate			multivariable		
			*p*-value	OR	95% CI	*p*-value	OR	95% CI
Maternal age (years, mean)	28.9	27.3	< 0.001	1.06	1.03–1.10	< 0.001	1.07	1.03–1.11
Maternal height (cm, mean)	163.3	165.8	< 0.001	0.94	0.91–0.96	< 0.001	0.93	0.90–0.95
Maternal pre-pregnancy weight (kg, median)	64.0	61.5	0.012	1.02	1.01–1.03			
Maternal pre-pregnancy BMI (kg/m^2^) (median)	24.1	22.6	< 0.001	1.10	1.06–1.14	< 0.001	1.10	1.06–1.14
Diabetes (GDM or DM) (%)	17.6	7.9	0.001	2.47	1.48–4.13			
GDM, diet treatment	10.8	5.3						
GDM, medical treatment	5.8	2.3						
Chronic DM	1	0.3						
Infertility treatment (%)	12.2	6.8	0.033	1.89	1.05–3.39			
IVF/ICSI	7.0	3.6						
Ovarian stimulation	3.8	1.8						
Insemination	1.4	1.4						
Chronic disease (%), excl. DM, HT	33.8	24.2	0.010	1.60	1.12–2.29	< 0.001	1.52	1.02–2.25
Hypertension (HT) (%)^a^	14.5	11.9	0.347	1.26	0.78–2.02			
Gestational HT	8.8	7.0						
Pre-eclampsia	6.8	5.6						
Chronic HT	0.7	1.0						
Fear of childbirth (%)	5.7	5.0	0.67	1.17	0.57–2.38			

^a^*8* primiparas, who had chronic or gestational *HT* had also pre-eclampsia

Intrapartum factors possibly associated with acute CS due to labor dystocia are presented in Table 2. In multivariable analysis, longer gestational age, less advanced cervical status at admission, chorioamnionitis and malposition remained as independent risk factors of CS. The great majority of malpositions were occipitoposterior positions (89%). The most common indications for induction of labor (IOL) were post term pregnancy (26.8% of inductions) and rupture of membranes with no contractions within 24 h of PROM (27.6%).

**Table 2:** Intrapartum factors according to delivery route

	CS (*n* = 296)	VD (*n* = 302)	univariate			multivariable		
			*p*-value	OR	95% CI	*p*-value	OR	95% CI
Gestational age (days, median)	285	281	< 0.001	1.06	1.04–1.08	< 0.001	1.08	1.03–1.12
Spontaneous contractions at admission (n)	106	195	< 0.001	0.30	0.21–0.42			
PROM (n)	101	66	0.001	1.85	1.29–2.67			
IOL (PG or balloon) (n)	90	42	< 0.001	2.71	1.80–4.07			
Indication for IOL								
Postterm pregnancy	28	7						
PROM	22	15						
Pre-eclampsia or HT	16	7						
Diabetes	6	6						
LGA	7	0						
SGA	0	2						
Other	11	5						
Cervix dilatation at admission (mean, cm)	1.5	2.6	< 0.001	0.57	0.48–0.67	0.002	0.65	0.50–0.85
Head position in birth canal at admission (mean, cm)	−2.7	−2.1	< 0.001	0.47	0.36–0.60	0.003	0.56	0.39–0.82
Chorioamnionitis (n)	95	10	< 0.001	10.9	5.01–23.6	< 0.001	21.3	6.86–65.9
Fetal malposition (n)	148	7	< 0.001	42.0	19.2–91.9	< 0.001	70.9	24.2–207
Oxytocin during labor (n)	281	220	< 0.001	6.98	3.92–12.5			
Amniotomy in labor (n)	129	148	0.183					
CTG abnormality (n)	123	12	< 0.001	17.3	9.28–32.2			
Fetal blood sample (n)	103	15	< 0.001	10.2	5.78–18.0			
Meconium-stained fluid (n)	101	40	< 0.001	3.31	2.20–5.00			
Use of IUPC (n)	202	10	< 0.001	63.4	32.2–125			
Epidural analgesia (n)	280	214	< 0.001	7.20	4.10–12.6			
PCB (n)	65	102	0.001	0.55	0.38–0.79			
Duration of delivery (mins, median)	960	620	< 0.001	1.19	1.14–1.23			

PROM = premature rupture of membranes

IOL = induction of labor

LGA = suspected Large for Gestational Age

SGA = suspected Small for Gestational Age

IUPC = intrauterine pressure catheter

PCB = paracervical block

The multivariable *p*-values and ORs are from logistic regression analyses carried out among the factors related to dystocia, i.e. the first eight main factors in the table

Neonatal outcome was good in both groups. Newborns in the CS group were slightly heavier than those in the control group (3655 g vs. 3503 g; OR 1.11 [1.07–1.15]). 94% of newborns whose birthweight was over 4500 g, were born by CS. There were five newborns, whose birthweight was under 2500 g, two of them in CS group and three were born vaginally. In CS group newborns had a higher umbilical artery pH (7.33 vs. 7.24; OR 1.11 [1.08–1.15]) but slightly poorer 5-min Apgar scores (8.81 vs. 8.88; OR 0.56 [0.41–0.77]). Admission to the pediatric care unit was low in both groups (5.7% vs. 5.0%, NS). Fetal scalp blood sample was taken during 19.6% of labors.

Although the parturients with abnormal CTG findings as a primary reason for CS were excluded from the study, CTG pathology was a secondary finding in many CS cases. To avoid an adverse effect on the results, we performed a subgroup analysis, where we removed parturients with any abnormal CTG findings. The results in univariate and multivariable analysis were otherwise similar with the original study group except for maternal age which did not differ significantly between the subgroups.

**Fig. 2:**
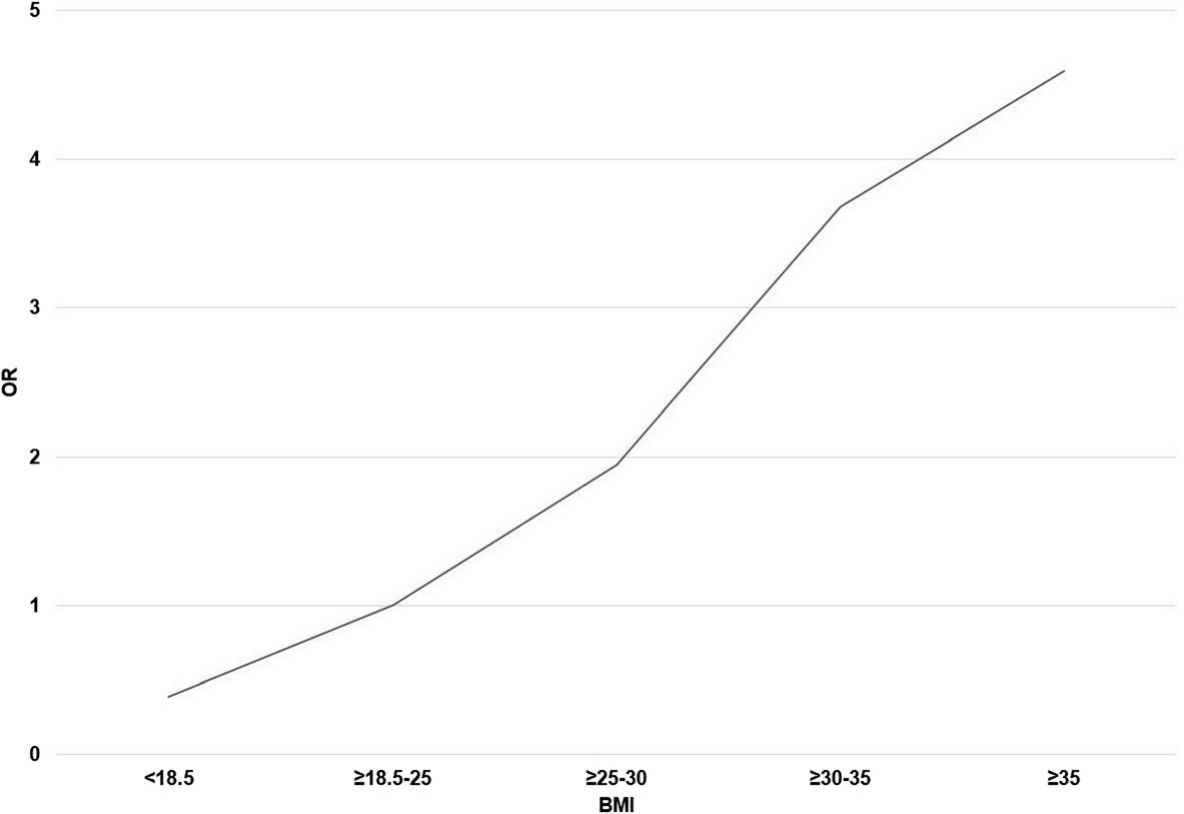
Influence of BMI on the risk of cesarean section due to labor dystocia

Significant differences were found between the study groups as regards pre-pregnancy BMI (Tables 3 and 4). Most parturients (64.2%) were of normal weight before pregnancy and in this BMI class the women experienced more VDs than CSs. When BMI was above normal, the risk of dystocia and intrapartum CS increased almost linearly (Fig. 2). Severely obese parturients (BMI ≥ 35 kg/m^2^) needed 4 hours longer to achieve successful VD when compared with normal-weight primiparas and almost 6 hours longer when compared with underweight primiparas (BMI < 18.5 kg/m^2^). Among moderate obese (BMI ≥30–35) primiparas, the most common indication for induction was post term pregnancy (*n* = 6; 42.9%), and among severe obese primiparas it was hypertension (including pre-eklampsia, *n* = 5; 33.3%).

**Table 3:** Route of delivery according to WHO BMI classes

BMI	Total		CS (291)		VD (299)		univariate			multivariable		
	*n*	%	*n*	%	*n*	%	*p*	OR	95% CI	*p*	OR	95% CI
< 18.5	22	3.7	5	1.71	17	5.69	0.073	0.39	0.14–1.09	0.791	0.82	0.20–3.44
≥18.5–25	379	64.2	162	55.7	217	72.6		1		0.011	1	
≥25–30	113	19.2	67	23.0	46	15.4	0.002	1.95	1.27–2.99	0.262	1.41	0.78–2.55
≥30–35	45	7.6	33	11.3	12	4.01	< 0.001	3.68	1.85–7.35	0.005	3.36	1.44–7.84
≥35	31	5.3	24	8.25	7	2.34	0.001	4.59	1.93–10.9	0.016	3.82	1.29–11.3

8 BMIs were missing

The multivariable *p*-values and ORs are from logistic regression analyses carried out among the most marked factors affecting delivery route, i.e. BMI class, maternal age, diabetes, chronic disease, induction of labor, chorioamnionitis, fetal malposition, gestational age, birth weight

**Table 4:** Maternal characteristics and intrapartum factors according to BMI WHO classification in the whole study population

BMI	< 18.5 (22)	≥18.5–25 (379)	≥25–30 (113)	≥30–35 (45)	≥35 (31)	*p*-value
Maternal age (y, mean)	24.5	27.9	28.6	28.8	28.9	0.007
Diabetes (GDM or DM) (%)	0	7.1	18.6	24.4	51.6	< 0.001
Chronic disease (%)	27.3	27.4	27.4	35.6	41.9	0.396
Thyroid disorder (%)	0	4	7.1	2.2	16.1	0.039
Gestational age (d, median)	281	283	285	289	286	^a^
Infertility treatment %	5	8.1	12.8	9.3	14.3	0.264
Admission dilatation (mean, cm)	2.8	2.2	1.9	1.8	1.4	0.007
Head position in birth canal at admission (mean, cm)	−1.9	−2.2	−2.6	−2.6	−2.8	< 0.001
Spontaneous contractions (%)	77.3	56.4	38.1	33.3	32.3	< 0.001
Induction of labor (%)	9.1	17.4	29.2	31.1	45.2	< 0.001
Duration of labor in VD excl. CS (h:min, median)	8:50	10:20	10:31	10:39	14:35	0.51
Oxytocin during labor (%)	68.2	81.5	89.4	93.3	87.1	0.024
Chorionamnionitis (%)	4.5	15.3	19.5	20.0	35.5	0.002
Fetal malposition (%)	13.6	23.8	31.9	28.9	38.7	0.113^b^
Use of IUPC (%)	18.2	29.1	42.5	57.8	61.3	< 0.001
PROM (%)	13.6	27.2	30.0	29.5	41.9	0.23^c^
Time between ROM and delivery (h:min)	4:05	8:02	8:50	8:47	11:40	^d^
CTG pathology	9.1	23.0	24.8	22.2	22.6	0.878
Birth weight (g, mean)	3245	3518	3667	3816	3718	< 0.001
LGA > 4500 g (%)	0	2.1	2.7	11.1	3.2	
SGA < 2500 g (%)	4.5	1.1	0	0	0	
5 min Apgar	8.86	8.85	8.90	8.93	8.39	0.307
Umb. artery pH	7.28	7.28	7.30	7.31	7.28	NS

^a^*p*-value was significant (< 0.05) when normal BMI (18.5–25) was compared to BMI group ≥ 25–30 or ≥ 30–35

^b^Significant trend (*p* = 0.014)

^c^Significant trend (*p* = 0.046)

^d^*p*-values between BMI groups < 18.5– ≥35 *p* = 0.001 and ≥ 18.5–25 *p* = 0.023

Premature rupture of membranes (PROM) was associated with obesity. In severely obese parturients (BMI > 35 kg/m^2^) PROM preceded labor in 41.9% of cases, while PROM occurred in 13.6 and 27.6% of cases in BMI classes of < 18.5 and 18.5–25 kg/m^2^, respectively – this trend was significant (*p* = 0.046). Obesity did not affect newborn outcome. In multivariable analysis, obesity remained as an independent risk factor of labor dystocia when BMI was 30 kg/m^2^ or more.

## Discussion

In line with the results of previous studies, advancing maternal age [3, 5, 6, 14, 15], high BMI [6, 7, 14–16] and maternal chronic disease [6, 17] were independently associated with labor dystocia and intrapartal CS in our study. Our finding of short stature being associated with dystocia has been shown in previous studies [15, 18], but there are also converse results [14].

The age of primiparas has risen during the last few decades, which may be one reason for the increased CS rate worldwide. In Finland the mean age of all primiparas was 28.6 years in 2014 [19], which is slightly lower than the mean age of the dystocia group in our study. Surprisingly, fear of childbirth did not increase the incidence of intrapartum CS for labor dystocia in our study population. In a Swedish study, FOC was associated with an increased emergency CS rate even after psychiatric counseling [20]. On the other hand, in a Finnish randomized trial, when fear of childbirth was treated in a psychoeducational group, treatment decreased the emergency CS rate [21]. In our study population, the incidence of FOC was in line with the national prevalence of FOC. This prevalence may reflect Finland’s health care system’s aim to recognize and treat parturients with FOC early in pregnancy. Besides these background factors, several intrapartum factors were also found to differ between groups, some of which may be regarded rather as a consequence of dystocia than a cause of it.

The results of the present study confirmed previous findings that admission to hospital at an early stage of labor, and induction of labor, are related to intrapartal CS [6, 15]. However, the indication for induction, such as prolonged pregnancy, rather than induction itself, may carry a risk of dystocia. Likewise, admission to hospital at an early stage of cervical dilatation [14, 15] may be a result of a diminished capacity of labor to proceed in some cases.

Oxytocin, intrauterine pressure catheters and epidural analgesia are used in the treatment of prolonged labor, which probably explains their greater use in the CS group. In the dystocia group, there was a greater need of analgesia, such as epidural analgesia, opiates and nitrous oxide, and many parturients needed several modes of analgesia. On the other hand, in successful VD, there was significantly more use of paracervical blocks. Our intrapartal findings support the clinical experience that prolonged labor is a sum of many factors, and it is hard to say which is first: lack of progress, fetal malposition, or chorioamnionitis.

The increased risk of labor dystocia brought about by obesity may be direct or the consequence of associated risk factors. In our multivariable test, mild obesity [BMI 25–30 kg/m^2^] did not appear to be an independent risk factor, but beyond that, BMI independently increased the risk of CS almost fourfold. As a new finding in our study, we noticed that among obese women PROM preceded labor more often than in normal-weight parturients. In addition, in line with the results of previous studies, overweight women came to hospital at an earlier stage of cervical dilatation, they less frequently had spontaneous contractions before admission to hospital, their labors were more often induced and they more often needed oxytocin during labor [6–8, 15, 16]. The reason for these findings is obscure, but they may reflect relative myometrial inactivity related to the unfavorable metabolic circumstances in obese parturients [22], possibly associated with endothelial dysfunction [22, 23]. It has also been speculated that oxytocin receptors might be influenced by maternal obesity [8].

Successful vaginal deliveries of obese parturients lasted 4 hours longer than among normal-weight parturients. This is in line with the results of previous studies, which have suggested that obese parturients need 2 hours more to progress in the first stage of labor, especially before 7 cm dilatation [8, 24]. It can be speculated that in our study population some severely obese women could have achieved vaginal delivery if they had been given more time in the first stage of labor.

A strength of our study is that the fairly large study material from a single center is homogeneous. The data on maternal background and deliveries is reliable and of good quality, being collected from the patients’ medical records. In the literature we found only one case-control study of labor abnormalities [25], and none regarding the risk of dystocia among term primiparas [14].

A limitation of the study is that diagnoses such as chorioamnionitis, fetal malposition and CTG pathology may not always have been registered in connection with successful VD. As this study was retrospective, we could not control the indication for CS and the diagnosis of dystocia in the delivery room. Dystocia is often a complex, multifactorial phenomenon. Hence, there is an unavoidable challenge as regards causality.

## Conclusions

Because CS can be problematic for the parturient and newborn, and also in regard to subsequent pregnancy and delivery, it is worth trying VD. Clinical possibilities to avoid dystocia are limited. Maternal pre-pregnancy BMI is one of the contributors that could be influenced before pregnancy. This way, clinicians could try to avoid dystocia and prevent CS. To prevent CS further, primiparas should be encouraged to delay admission to hospital until the cervix is properly dilated and IOL of primiparas with an unfavorable cervix should be avoided. When there is an obese parturient in the delivery room, clinicians should recognize this risk factor and allow considerably more time in the first stage of labor before diagnosing dystocia.

List of abbreviations: CS, cesarean section; VD, vaginal delivery; BMI, body mass index; CTG, cardiotocography; GDM, gestational diabetes mellitus; FOC, fear of childbirth; IOL, induction of labor; PROM, premature rupture of membranes.
